# A Novel Ternary Catalyst PW_4_@MOF-808@SBA-15 for Deep Extraction Oxidation Desulfurization of Model Diesel

**DOI:** 10.3390/molecules29174230

**Published:** 2024-09-06

**Authors:** Yan Gao, Shuai Huang, Nina Han, Jianshe Zhao

**Affiliations:** 1 Department of Chemistry, Xinzhou Normal University, Xinzhou 034000, China; 2 College of Chemistry & Materials Science, Northwest University, Xi’an 710069, China

**Keywords:** polyoxometalate, MOF-808, mesoporous SBA-15, extraction oxidation desulfurization, chemical process

## Abstract

In this work, a novel heterogeneous catalyst consisting of peroxophosphotungstate, microporous MOF-808, and mesoporous SBA-15 was synthesized, characterized, and used to remove sulfides from model fuel. The prepared material, PW_4_@MOF-808@SBA-15, exhibits excellent catalytic activity with a desulfurization efficiency of 99.8% in 60 min for multicomponent simulated fuel, and the desulfurization rate can reach more than 90% after ten consecutive cycles. The excellent catalytic activity and reusability are attributed to the hierarchically porous hybrid material MOF-808@SBA-15, which can effectively encapsulate PW_4_ and provide a site for the oxidation of sulfides.

## 1. Introduction

With the continuous growth in the global demand for energy, the environmental problems caused by the massive use of traditional petrochemical energy have gradually become prominent [1,2]. Among them, problems such as air pollution and acid rain caused by the sulfur oxides emitted from fuel combustion are particularly prominent [3,4]. In view of the environmental issues caused by the combustion of sulfur-containing compounds in liquid fuels, strict environmental laws have been enacted around the world to limit the sulfur content in fuels, such as below 10 ppm for diesel [5,6]. There are many kinds of sulfides in fuel, which can be divided into two categories: active sulfur species (elemental sulfur, hydrogen sulfide, mercaptans) and inactive sulfur species (aromatic sulfides and heterocyclic sulfides) [7,8]. Among them, the removal of refractory organic sulfur compounds such as dibenzothiophene and its derivatives is challenging. Therefore, it is necessary to find efficient and economical desulfurization methods. Traditional hydrodesulfurization technology (HDS) is a method of removing sulfides from fuel, which converts organic sulfur compounds to hydrogen sulfide and hydrocarbons by reacting with hydrogen under the action of a catalyst [9,10,11]. However, HDS can only effectively remove the active sulfides and part of the inactive sulfides, and reaction conditions are harsh, which led to a burst of research on alternative methods such as extraction oxidation desulfurization technology (EODS) [12,13,14]. Pure extractive desulfurization (EDS) is a method to extract sulfide from fuel oil by using the difference in the solubility of sulfide in specific solvents and fuel oil. Oxidative desulfurization (ODS) can convert the organic sulfide in fuel into a more polar substance, and then the more polar substance is further separated from the fuel by extraction or adsorption to achieve the purpose of reducing the sulfur content. EODS is a combination of extraction and oxidation processes, in which sulfides are first extracted into the extraction phase and then oxidized to sulfoxides and/or sulfones in the presence of an oxidant and a catalyst. The process continues until a low-sulfur or even sulfur-free fuel is obtained [15,16]. EODS can be carried out with an oxidant and without any catalyst but is generally less effective and requires extremely harsh reaction conditions such as high temperature and pressure. Therefore, highly active and environmentally friendly catalysts are needed for the efficient removal of refractory sulfides through EODS. Common oxidants include O_2_, O_3_, H_2_O_2,_ and tert-butyl peroxide, among which H_2_O_2_ is the most widely used oxidant during the EODS process because it is cheap, easily available, and environmentally friendly, and has a high active oxygen content [17,18,19,20]. Several metal-containing substances, including metal oxide, polyoxometalates (POMs), metal complexes, and metal–organic frameworks, have been applied to EODS [21,22,23]. In particular, polyoxometalates have become attractive candidates for EODS in recent years due to their simple synthesis, stable structure, and adjustable structure and properties [24]. Polyoxometalates (POMs) with peroxy bonds exhibit higher catalytic activity in EODS than traditional Keggin-type polyoxometalates [25,26]. This is due to the higher electrophilicity of some of the oxygen atoms in peroxopolyoxometalate, which can effectively improve the EODS efficiency. Salete et al. [26] prepared Keggin-type phosphotungstates modified with different cations ([BPy]_3_PW_12_, [BMIM]_3_PW_12,_ and [HDPy]_3_PW_12_), which effectively removed several refractory sulfides from fuel using [BMIM]PF_6_ as the extraction agent. Meanwhile, the formation of peroxy complexes (PW_x_O_y_), which play a key role in the catalytic reaction, was verified through ^31^P NMR. In view of the above conclusions, the authors prepared peroxophosphotungstate (nBu_4_N)_3_{PO_4_[WO(O_2_)_2_]_4_} for fuel desulfurization, and the results showed that the catalyst could achieve a high desulfurization rate under more economical and energy-saving conditions [25]. Shi and co-workers [27] also prepared heterogeneous catalyst SiO_2_-BisILs [(PW_12_O_40_)^3−^] assembled from peroxophosphotungstate and ionic liquid brush via EODS, and the prepared material showed excellent catalytic activity for dibenzothiophene (DBT). Although POMs show superior activity in EODS, they cannot be separated from fuel due to their high solubility and cannot be reused continuously. Encapsulating POMs in solid materials with a large specific surface area and suitable pore structure is an effective way to solve the above shortcomings.

Metal–organic frameworks (MOFs), a class of ordered crystalline compounds, have become promising contemporary materials due to their tunable chemical functionalities, high specific surface area, and pore volume [28]. The development of new hybrid systems, which can combine the advantages of each component, has been attracting a large number of researchers. Some researchers have tried to combine MOFs with different materials, such as materials with two-dimensional structures (carbon nanotubes, graphene, and boron nitride) or materials with three-dimensional pore structures (MCM-41 and SBA-15), and so on. These MOF-based composites not only combine the advantages of the two materials but also show unique properties. Tapas Kumar Maji and Anindita Chakraborty [29] prepared a hybrid Mg-MOF-74@SBA-15 material through immobilizing Mg-MOF-74 nanocrystals into the mesopores of SBA-15, which has remarkable CO_2_ adsorption capacity at room temperature due to its unique pore structure. Dirk E. De Vos and co-workers [30] obtained a hybrid catalyst, (Zr)UiO-66(NH_2_)/SBA-15, by means of “solid-state” crystallization, i.e., the selective growth of (Zr)UiO-66(NH_2_) nanocrystals in mesoporous SBA-15, and the authors noted that this hybrid material, (Zr)UiO-66(NH_2_)/SBA-15, has higher catalytic activity and mechanical stability than pure MOFs.

MOF-808, as one of the {Zr_6_O_8_}-cluster-based MOFs, has attracted extensive attention due to its high hydrophilic porous surface, good biocompatibility, large cavity, and pore volume. However, the poor stability of MOF-808 has become the main factor restricting its large-scale commercial application. Mesoporous molecular sieve SBA-15 has attracted much attention from researchers due to its highly ordered mesoporous structure, good thermal stability, and high mechanical strength [31]. The hierarchically porous composite constructed by MOF material and mesoporous molecular sieve SBA-15 has a rich chemical environment and stable mechanical properties, which can avoid the disadvantages of MOF materials and provide more possibilities for the application of MOFs [29,30,32].

In this work, a more robust heterogeneous catalyst was constructed by combining peroxophosphotungstate [(C_4_H_9_)_4_N]_3_{PO_4_[WO(O_2_)_2_]_4_}, marked as (PW_4_), and hierarchically porous composite MOF-808@SBA-15 through a simple hydrothermal strategy, which was used for the removal of sulfides from model fuel. The structure of the target product was analyzed by various characterization methods, and then the desulfurization performance was studied using the multicomponent model fuel.

## 2. Results and Discussion

### 2.1. Characterization

**Figure 1:**
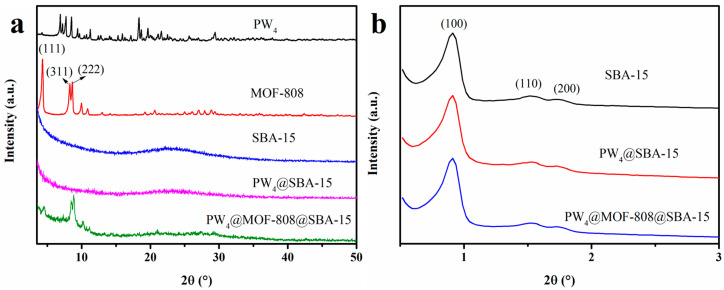
XRD patterns of the obtained materials in 2Θ ranges from 3 to 50° (**a**) and 0.5 to 3° (**b**).

The crystal structures of the prepared materials were analyzed by powder XRD, as depicted in Figure 1. The obtained pattern for PW_4_ shows several characteristic peaks at 7–30°. For pure MOF-808, many strong peaks are shown at 2θ values of 4.3, 8.3, and 8.7°, which were assigned to the characteristic planes (111), (311), and (222). Tri-peaks were observed at low angles in the SBA-15 pattern, which were labeled as p6mm hexagonally symmetrical (100), (110), and (200) reflections, confirming the successful preparation of SBA-15. After incorporating PW_4_, no significant change was found in the characteristic peak, indicating that PW_4_ was uniformly dispersed in the SBA-15 channel. For PW_4_@MOF-808@SBA-15, the presence of the same signals from MOF-808 confirmed the successful incorporation of MOF-808 into SBA-15, and the absence of signals from PW_4_ indicated uniform encapsulation of PW_4_. Furthermore, the low-angle peaks in the two composites (PW_4_@SBA-15 and PW_4_@MOF-808@SBA-15) were similar to those of SBA-15, indicating that the ordered mesoporous pores corresponding to SBA-15 were not destroyed.

**Figure 2:**
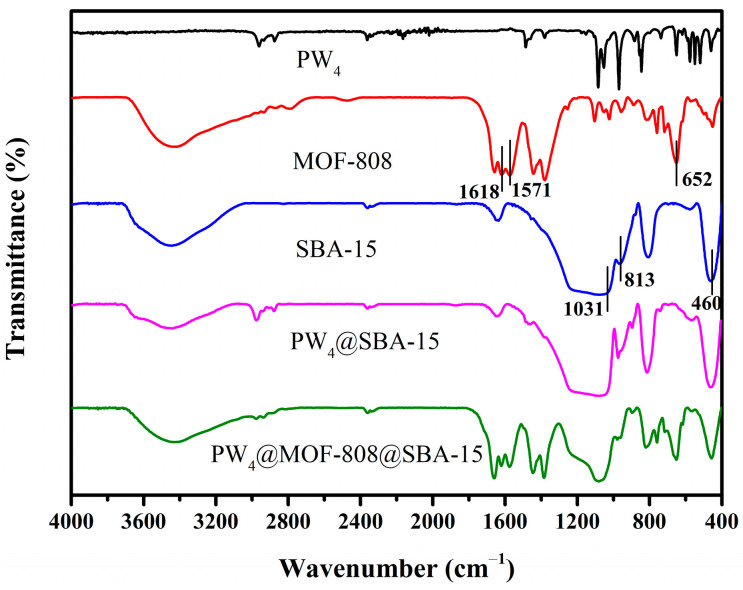
FT-IR spectra of the obtained materials.

The IR spectra of the raw materials (PW_4_, MOF-808, and SBA-15) and two composites (PW_4_@SBA-15 and PW_4_@MOF-808@SBA-15) were recorded and are displayed in Figure 2. For PW_4_, several intense bands were observed at the range of 849–1083 cm^−1^, which is attributed to the Venturello structure anion {PO_4_[WO(O_2_)_2_]_4_}^3^. For MOF-808, the strong peak at 652 cm^−1^ is attributed to the stretching vibration of Zr-O, and two absorption bands are shown at 1618 and 1571 cm^−1^, corresponding to COO^-^ asymmetric stretching. In the case of SBA-15, the strong absorption peaks at 1031, 813, and 460 cm^−1^ are attributed to the asymmetric and symmetrical tensile vibration and bending patterns of the Si-O-Si frame. The FT-IR spectrum of PW_4_@SBA-15 shows not only the characteristic peaks belonging to SBA-15 but also some characteristic peaks belonging to PW_4_. In the FT-IR spectrum of PW_4_@MOF-808@SBA-15, the same absorptions as those of SBA-15 and MOF-808 were observed, with a slight shift, indicating the successful immobilization of MOF-808, and the peaks attributed to PW_4_ were not witnessed, indicating the homogeneous dispersion of PW_4_ within the pore structure.

**Figure 3:**
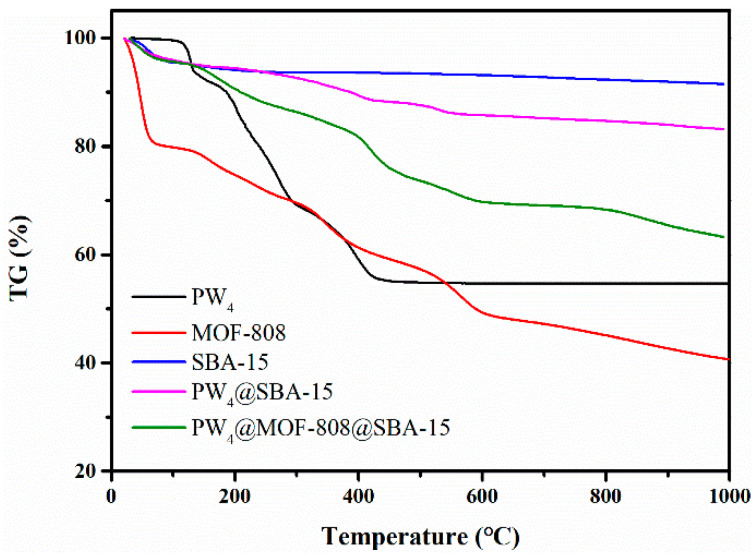
Thermogravimetric curves of the obtained materials.

The trends in the thermogravimetric curves of SBA-15 and PW_4_@SBA-15 were similar except for the weight loss rate, which verified the presence of the active component PW_4_ in PW_4_@SBA-15 (Figure 3). The thermogravimetric curve for PW_4_@MOF-808@SBA-15 shows a similar profile to that of MOF-808. After the weight loss of water and solvent, two significant weight losses occurred between 258 °C and 590 °C, which correspond to the removal of BTC and Zr clusters.

**Figure 4:**
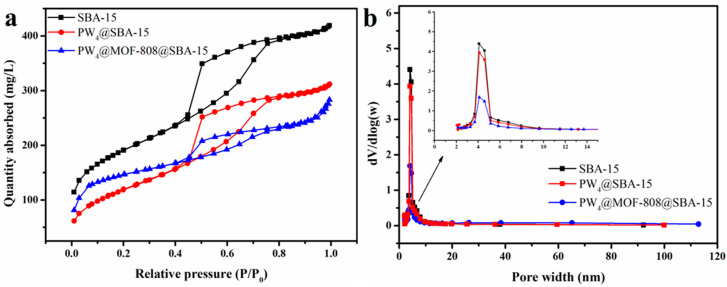
N_2_ adsorption–desorption isotherms (**a**) and pore size distribution (**b**) of the obtained materials: SBA-15, PW_4_@SBA-15, and PW_4_@MOF-808@SBA-15.

**Figure 5:**
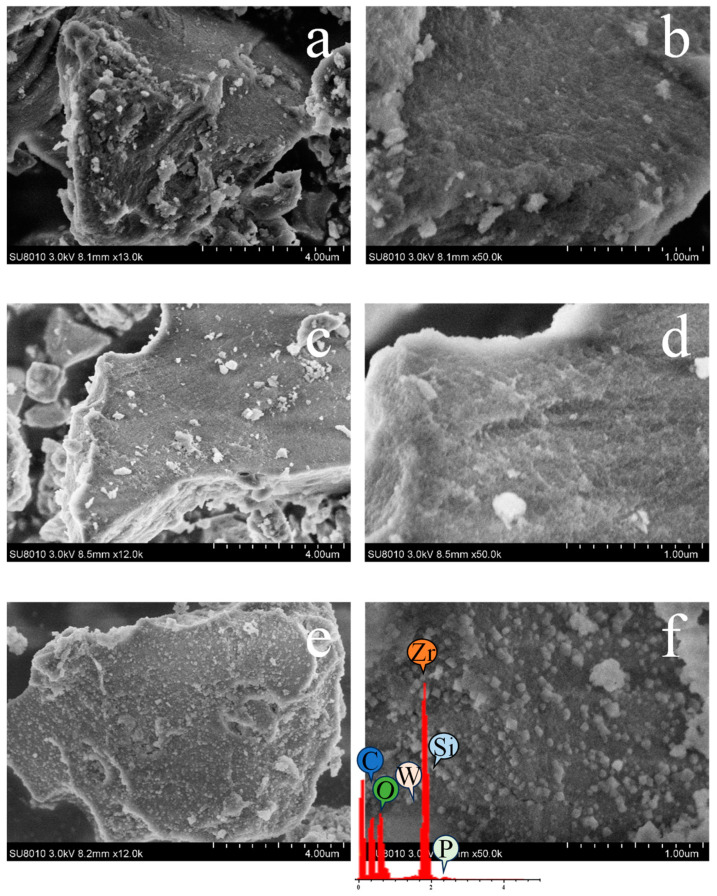
SEM images of the SBA-15 (**a**,**b**), PW_4_@SBA-15 (**c**,**d**), and PW_4_@MOF-808@SBA-15 (**e**,**f**) at different magnifications, and energy-dispersive X-ray spectroscopy (EDS) spectrum of PW_4_@MOF-808@SBA-15.

The porous properties of pure SBA-15, PW_4_@SBA-15, and PW_4_@MOF-808@SBA-15 were determined through N_2_ adsorption–desorption experiments, and the test results are shown in Figure 4 and Table 1. The isotherm of the SBA-15 sample was a typical type IV with an H1-type hysteresis loop, indicating the presence of mesoporous pores. And the pore size distribution of the original SBA-15 was narrow, mainly in the range of 4.1–4.6 nm. The isotherm type of composite PW_4_@SBA-15 is was same as that of the original SBA-15, indicating that the original pore structure was preserved after loading PW_4_. The isotherm of PW_4_@MOF-808@SBA-15 exhibited a combination of type I (characteristic of microporous materials) and type IV (characteristic of mesoporous materials). That is, composite PW_4_@MOF-808@SBA-15 showed the presence of additional micropores compared to the mesoporous pores of the original SBA-15. Compared with pure SBA-15, the specific surface area and empty volume of PW_4_@SBA-15 decreased, caused by the occupation of PW_4_ in the channels. And the specific surface area and empty volume of PW_4_@MOF-808@SBA-15 had more obvious decreases, indicating that MOF-808 nanocrystals were partially filled in the channels. Although the SEM images (Figure 5) show the presence of few MOF-808 nanocrystals on the surface of SBA-15, the reduced adsorption amount and partial filling of the mesopores of SBA-15 confirmed that the channels of SBA-15 could serve as sites for the growth of MOF-808 nanoparticles [29]. In addition, it was possible to confirm that the channels of SBA-15 were occupied by comparing the size distribution plot.

The morphologies SBA-15, PW_4_@SBA-15, and PW_4_@MOF-808@SBA-15 were observed from SEM images (Figure 5). The SEM images show that SBA-15 was an irregular mass, and the morphology of PW_4_@SBA-15 had no significant change after the introduction of PW_4_. Although the appearance of PW_4_@MOF-808@SBA-15 was similar to that of pure SBA-15, the formation of MOF-808 on the surface of SBA-15 could be clearly seen. The chemical composition of PW_4_@MOF-808@SBA-15 was further evaluated using the EDS technique. The detection of characteristic elements P and W indicated the successful introduction of the active component PW_4_.

**Figure 6:**
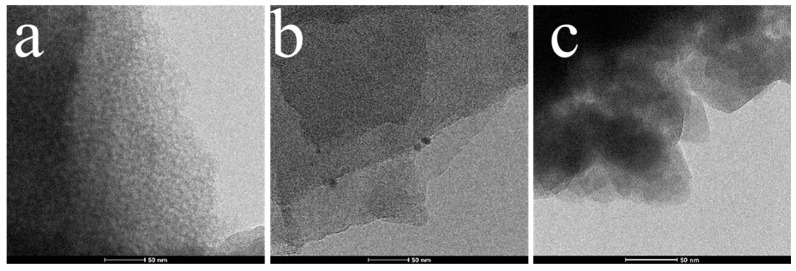
TEM images of SBA-15 (**a**), PW_4_@SBA-15 (**b**), and PW_4_@MOF-808@SBA-15 (**c**).

**Figure 7:**
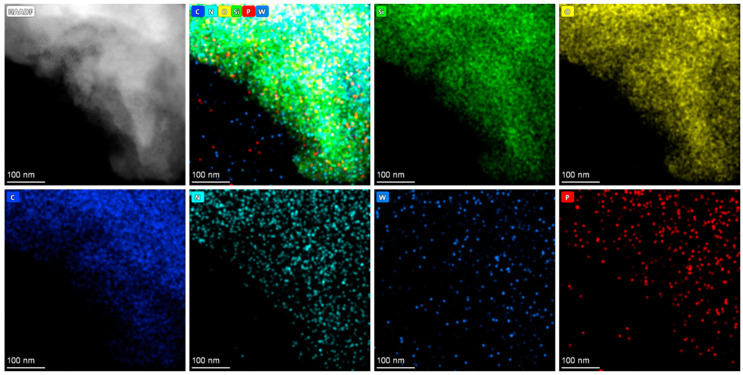
Elemental mapping of PW_4_@SBA-15.

**Figure 8:**
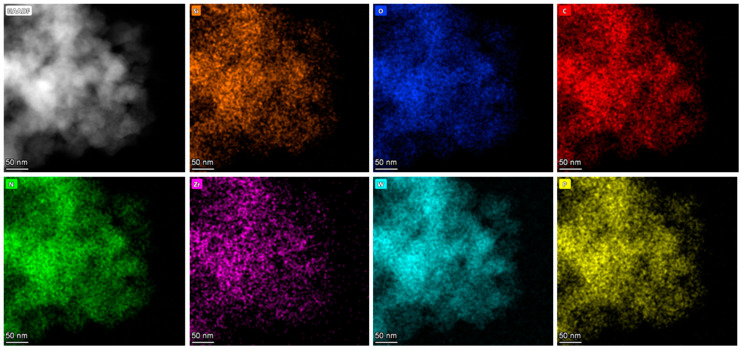
Elemental mapping of PW_4_@MOF-808@SBA-15.

The TEM image of PW_4_@SBA-15 shows clear areas and darker areas corresponding to empty holes and hole walls, which are similar to those of pure SBA-15 (Figure 6). At the same time, scattered black spots were observed in the pores, indicating that PW_4_ was evenly distributed in the pores of SBA-15. The TEM image of PW_4_@MOF-808@SBA-15 shows that the black spots were denser and larger, which may have been because the pore channels were occupied by MOF-808, and the octahedral MOF-808 could be seen on the edge of the sample, which is also consistent with the SEM results. Elemental mapping was used to confirm the homogeneous distribution of guests in the SBA-15 sample. For PW_4_@SBA-15, the uniform distribution of P and W elements attributed to PW_4_ indicated that the active components were evenly distributed in the pores (Figure 7). For PW_4_@MOF-808@SBA-15, the P and W elements attributed to the PW_4_ and Zr elements attributed to MOF-808 were uniformly distributed, which further indicated that the active component and MOF-808 existed in the SBA-15 channels (Figure 8).

**Figure 9:**
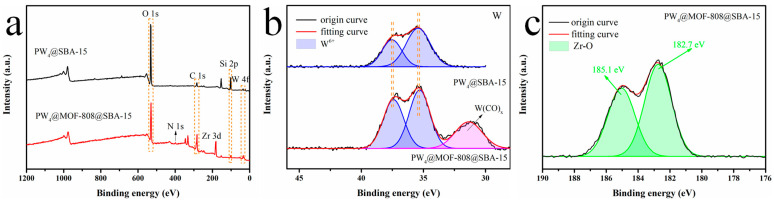
XPS spectra representing PW_4_@SBA-15 and PW_4_@MOF-808@SBA-15: (**a**) survey scan, (**b**) W4f, (**c**) Zr3d.

The composition and chemical environment of PW_4_@SBA-15 and PW_4_@MOF-808@SBA-15 were further elucidated by XPS (Figure 9). The survey scan of two composites, PW_4_@SBA-15 and PW_4_@MOF-808@SBA-15, revealed the presence of W 4f, C 1s, O 1s, N 1s, and P 2p elements, while Zr 3d was detected in PW_4_@MOF-808@SBA-15. The high-resolution spectra of W 4f for PW_4_@SBA-15 and PW_4_@MOF-808@SBA-15 are shown in Figure 9b. For PW_4_@SBA-15, the binding energies of 37.5 (W 4f_5/2_) and 35.4 eV (W 4f_7/2_) were assigned to the W(VI) oxidation state. For PW_4_@MOF-808@SBA-15, the negative chemical shift (ca. 0.16 eV) indicated the chemical interaction between PW_4_ and MOF-808 [33]. In addition, a peak of W(CO)x appeared at 31.4 eV, which further proved that the active ingredient PW_4_ may have been covalently linked to MOF-808 via the -COOH functional group on the organic linker [34]. Additionally, the high resolution of the Zr 3d spectrum of PW_4_@MOF-808@SBA-15 showed two peaks at 185.1 and 182.7 eV, which were assigned to Zr 3d_5/2_ and Zr 3d_3/2_.

### 2.2. Catalytic Performance

#### 2.2.1. EODS Performance of Different Catalysts

The desulfurization of the simulated diesel containing dibenzothiophene (DBT), 4-methyldibenzothiophene (4-MDBT), and 4,6-dimethyldibenzothiophene (4,6-DMDBT), which are the most common refractory sulfur compounds in liquid fuels, was carried out using MeCN as the extraction agent, H_2_O_2_ as the oxidant, and different materials as the catalyst. In the typical desulfurization process, a biphasic system consisting of 0.75 mL of simulated diesel and equal volumes MeCN and 3 umol PW_4_ or composite containing 3 umol PW_4_ were strongly stirred for 10 min under 70 ℃ to activate the initial extraction of sulfur compounds from the model diesel to the extraction solvent. Then, H_2_O_2_ (0.21 mmol, H_2_O_2_/S molar ratio = 6) was poured into the system to start the oxidation stage. Periodically, the upper fuel phase was sampled to analyze the variation in sulfide concentration.

**Figure 10:**
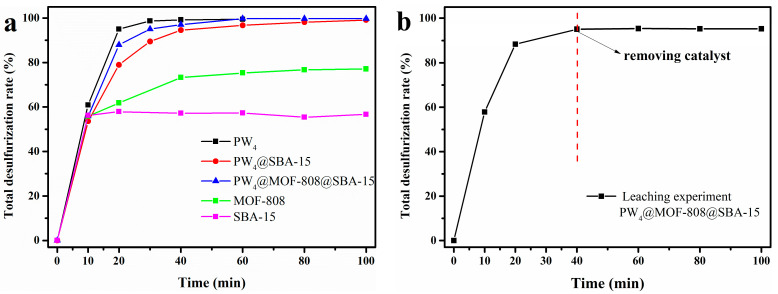
Desulfurization of the multicomponent model diesel catalyzed by different materials (**a**) and leaching experiments of the catalyst PW_4_@MOF-808@SBA-15 (**b**).

Pure PW_4_, as a homogeneous catalyst, demonstrated superior desulfurization performance, removing 99.2% of the sulfur in the simulated fuel within 40 min (Figure 10a). Although PW_4_ showed excellent catalytic activity in the catalytic oxidation of sulfides, it was difficult to recover from the reaction medium, which restricts its development in industrial applications. The heterogeneous catalyst prepared by filling PW_4_ in the SBA-15 channel for fuel desulfurization effectively improved the recyclability of the catalyst. The kinetic profile of the heterogeneous catalyst PW_4_@SBA-15 was similar to that of the homogeneous catalyst PW_4_, but only 98.9% of the sulfides was removed within an extended 100 min. Pure MOF-808 displayed moderate desulfurization activity with a sulfur removal rate of 77.1% in 100 min. Compared with pure MOF-808 and PW_4_@SBA-15, the ternary catalyst PW_4_@MOF-808@SBA-15 could remove sulfur compounds from fuel with higher efficiency, which was attributed to the uniform dispersion of PW_4_ molecules as a single active site and the synergistic action between PW_4_ and MOF-808. To verify that the active component PW_4_ was confined to the pore material, a leaching experiment was performed over the PW_4_@MOF-808@SBA-15 catalyst (Figure 10b). The results showed that the removal rate of sulfide remained basically unchanged after the catalyst filtration, indicating that the active substance was stably confined.

Considering the excellent catalytic activity of PW_4_@MOF-808@SBA-15 for model diesel, the ternary catalyst PW_4_@MOF-808@SBA-15 was used to desulfurize commercial diesel with a primary sulfur concentration of 1271 ppm. After treatment under the same conditions, the sulfur concentration of the commercial diesel was reduced to 105 ppm, corresponding to a 91.7% desulfurization rate. The desulfurization efficiency for commercial diesel was lower than that for simulated diesel, which may have been due to the more complex types of sulfides in real diesel.

#### 2.2.2. Reusability

**Figure 11:**
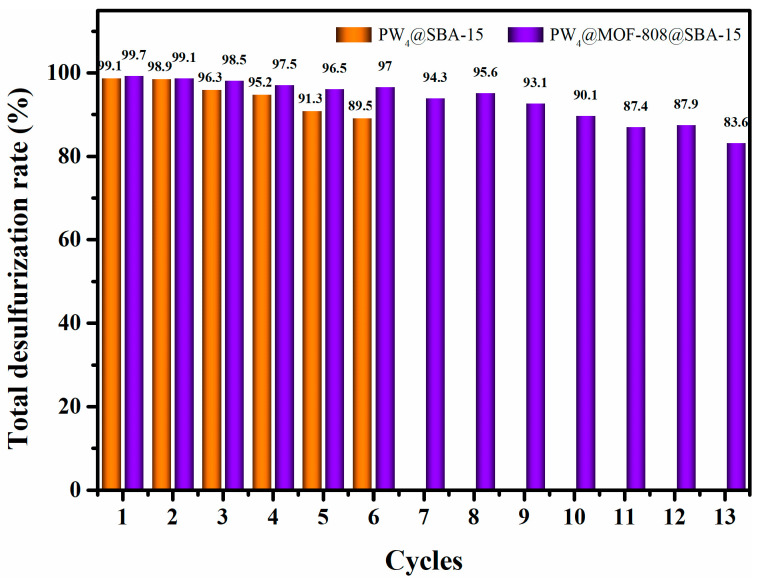
Reusability of heterogeneous catalysts PW_4_@SBA-15 and PW_4_@MOF-808@SBA-15.

The reusability of both heterogeneous catalysts PW_4_@SBA-15 and PW_4_@MOF-808@SBA-15 was measured in order to assess their practical application potential (Figure 11). The solid catalyst remained in the MeCN extraction layer during the reaction process. After a single test, the reused catalyst was centrifuged, washed, and dried, and then added to a new desulfurization system containing fresh simulated diesel, extractant, and oxidizer for the next ECODS cycle. The results showed that the desulfurization activity of PW_4_@SBA-15 decreased significantly after six consecutive cycles with a sulfide removal rate of 89.5%, which may have been due to the loss of PW_4_ adsorbed in the SBA-15 channel with the progression of the test. The reusability of the ternary catalyst PW_4_@MOF-808@SBA-15 was significantly better than that of composite PW_4_@SBA-15, which may have been due to the presence of MOF-808 inhibiting the loss of active component PW_4_.

### 2.3. Comparison with Other Catalysts

**Figure 12:**
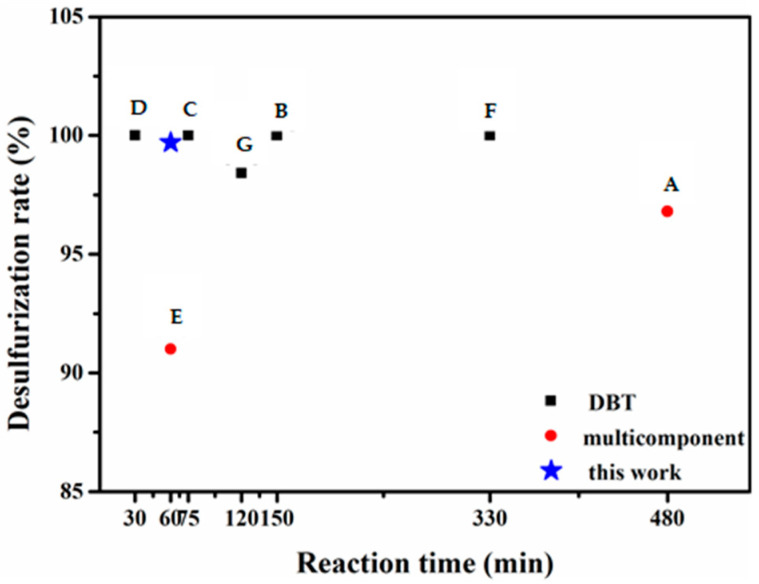
Desulfurization activity comparison (A [35], B [36] C [37], D [38] E [39], F [40] G [41]).

A comparison of the removal of organic sulfur substrates from model fuel catalyzed by the ternary catalyst PW_4_@MOF-808@SBA-15 prepared in this work and POM-based catalysts reported in the literature is given in Table 2 and Figure 12. Compared with similar catalysts (PMo_12_@MOF-808@SBA-15, SRL-POM@MOF-199@MCM-41, and POM-MOF@Fibercloth), the catalyst PW_4_@MOF-808@SBA-15 prepared in this work can remove a variety of sulfides from fuel in a shorter time, which is mainly due to the high activity of the active component PW_4_. Compared with simple composites, the catalyst PW_4_@MOF-808@SBA-15 has the advantage of high catalytic activity against a variety of sulfides under mild conditions.

### 2.4. Possible Mechanism

**Figure 13:**
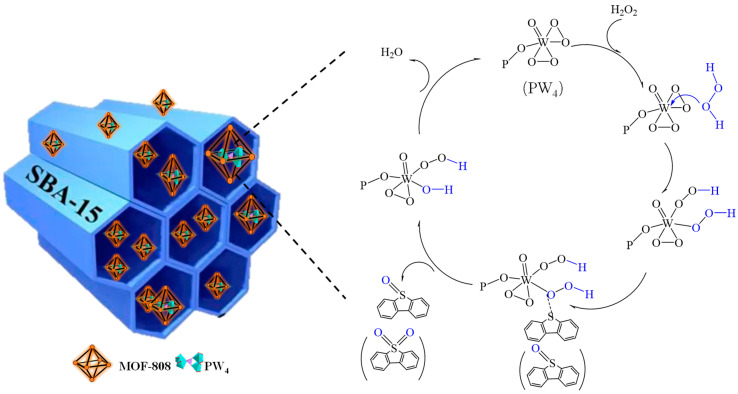
The proposed mechanism of EODS over the PW_4_@MOF-808@SBA-15 catalyst.

On the basis of the reported studies, a possible mechanism for the removal of organic sulfides from model fuel by EODS technology is proposed. The EODS system consists of an upper fuel phase and a lower extraction phase, and the catalyst PW_4_@MOF-808@SBA-15 exists in the lower extraction phase. At the initial stage of the reaction, the sulfides in the fuel phase were first extracted into the lower extractant, and the initial extraction rate was in the range of 53.6–60.8% (as shown in Figure 10). Then, the addition of the oxidizing agent H_2_O_2_ initiated the catalytic oxidation reaction of the sulfides in the extraction phase. While the sulfides were oxidized to large polar sulfones, more sulfides in the upper fuel phase were continuously transferred to the extraction layer until a low-sulfur or sulfur-free fuel was obtained. During the catalytic oxidation stage, PW_4_ in the PW_4_@MOF-808@SBA-15 catalyst combined with H_2_O_2_ to form an active species, which is a key intermediate in the oxidation reaction of POM-based catalysts with H_2_O_2_ as an oxidizing agent [42]. Under the action of the active species, the sulfides were oxidized to sulfoxides, then further oxidized to sulfones (Figure 13) [43].

## 3. Experimental Section

### 3.1. Chemicals and Reagents

All reagents used in this work were purchased from commercial suppliers without further purification. For the synthesis of materials, phosphotungstic acid (H_3_PMo_12_O_40_·nH_2_O, Bide, Shanghai, China, AR), H_2_O_2_ (Sigma-Aldrich, St. Louis, MO, USA, 30 wt%), tetrabutylammonium chloride (Bu_4_NCl, 99%, Bide), pluronic P123 (Aldrich, St. Louis, MO, USA, AR), tetraethyl orthosilicate (TEOS, Sigma-Aldrich, 98%), zirconium tetrachloride (ZrCl_4_, Aldrich, 99.5%), benzene tricarboxylic acid (H_3_BTC, Aldrich, 95%) were used. Dibenzothiophene (DBT, 99%, Aladdin. Bay City, MI, USA), 4-methyldibenzothiophene (4-MDBT, 96%, Aladdin), 4,6-dimethyldibenzothiophene (4,6-DMDBT, 97%, Aladdin), N-octane (99.9%, Aladdin), tetradecane (99%, Aladdin) were used to carry out desulfurization tests.

### 3.2. Catalyst Preparation

#### 3.2.1. Synthesis of PW_4_@SBA-15

Peroxophosphotungstate (Bu_4_N)_3_{PO_4_[WO(O_2_)_2_]_4_}, abbreviated as PW_4_, was obtained according to our previous work [44]. SBA-15 was obtained by the following experimental method: 1.5 g of pluronic P123 was poured into 56.3 mL of 2.4 M HCl aqueous under stirring at 40 °C, and then 3.3 g of TEOS was added to the above solution. The mixed solution was stirred for 24 h at 40 °C and then heated at 100 °C for 24 h. After cooling, the SBA-15 sample was obtained by calcining the isolated white solid for 5 h at 550 °C. PW_4_@SBA-15 was prepared by the impregnation method. Then, 1.0 g of SBA-15 was added to 5 mL of acetonitrile solution dissolved with 0.50 g of PW_4_ under magnetic stirring. After 24 h, the solid product, PW_4_@SBA-15, was collected and dried.

#### 3.2.2. Synthesis of PW_4_@MOF-808@SBA-15

PW_4_@MOF-808@SBA-15 was obtained through the solvothermal method [35,36], as follows: 0.50 g of SBA-15, 0.50 g of PW_4_, 0.51 g of ZrCl_4,_ and 0.16 g of H_3_BTC were poured into 25 mL of DMF and treated with ultrasound for 30 min. Then, the mixture was transferred to Teflon autoclaves and heated at 130 °C for 24 h. Finally, the solid was collected after cooling, washed with DMF and EtOH three times, and dried at 60 °C overnight; the yield was 63.5%.

Pure MOF-808 was synthesized by the same solvothermal method as PW_4_@MOF-808@SBA-15, without adding SBA-15 and PW_4_.

### 3.3. Catalyst Characterization

The functional group information of different samples was acquired through a Fourier-transform infrared (FT-IR) spectrometer (EQUINOX 55) using the KBr pellet method in the wavenumber range of 4000–400 cm^−1^ (Bruker, Ettlingen, Germany). The crystal structures of the prepared materials were acquired by powder X-ray diffraction (XRD) on a Bruker D8 Advance (Bruker, Ettlingen, Germany) and Shimadzu XRD-7000S (Shimadzu, Tokyo, Japan) at room temperature. The thermal stability of the prepared materials was analyzed on a NETZSCH STA 449A thermal analyzer (Netzsch-GeräTebau GmbH, Selb, Germany) from 30 to 1000 °C with a heating rate of 5 °C/min under a nitrogen atmosphere. The physical structure parameters of the porous materials were collected by a N_2_ adsorption–desorption apparatus on a JW-BK 200 at 77 K (CASIO, Tokyo, Japan), and all samples were degassed at 120 °C for 6 h before the test. The morphology and element composition of the samples were examined by a scanning electron microscope (SEM, SU8010, Hitachi, Tokyo, Japan) with electron energy-dispersive spectroscopy (EDX) at 3 kV. The element distribution of the samples was further examined by transmission electron microscopy (TEM) on a Talos F200X instrument (Thermo Fisher Scientific, Waltham, MA, USA). Before the test, about 5 mg of the powder sample was fully dispersed in 1 mL of ethanol under ultrasound and then dropped on the surface of the copper mesh. The surface chemical composition and electronic states of the materials were analyzed by X-ray photoelectron spectroscopy (XPS) on PHI-Veroprobe 5000 Ⅲ spectrometer (ULVAC-PHI, Maozaki, Japan) with an Al Kα X-ray source. The sulfide concentration during the reaction was periodically measured by a gas chromatograph (Agilent 7890, Agilent Technologies, Santa Clara, CA, USA) equipped with an HP-5 type (30 m × 0.25 mm) column; the temperatures of the inlet, detector, and oven were 150 °C, 250 °C, and 150 °C, respectively.

### 3.4. Evaluation of EODS Efficiency

A given amount of DBT, 4-MDBT, and 4,6-DMDBT was completely dissolved in n-octane to obtain a simulated diesel with a total sulfur content of 1500 ppm. EODS tests were executed in a closed glass container filled with simulated oil, acetonitrile as the extractant, and catalyst under continuous stirring. More precisely, 3 µmol PW_4_ or composite containing 3 µmol PW_4_, 0.75 mL of model diesel, and an equal volume of acetonitrile were added to the reactor in sequence and stirred for 10 min under 70 °C to achieve extraction equilibrium. Then, the oxidation process was initiated by adding 0.21 mmol of hydrogen peroxide to the mixture. During the whole process, the upper diesel phase was periodically sampled, and the sulfide content was quantitatively analyzed by the internal standard method for gas chromatography.

### 3.5. Catalyst Recovery

After the reaction, the mixture was centrifuged for 10 min to recover the solid catalyst. Then, the catalyst was fully washed with octane and acetonitrile and dried overnight at 60 °C. The recovered catalyst was added to the fresh EODS system to re-evaluate its desulfurization performance. Several consecutive tests were carried out to assess its reusability.

## 4. Conclusions

A novel ternary catalyst, PW_4_@MOF-808@SBA-15, was successfully prepared through the facile solvothermal method in the presence of mesoporous SBA-15 and active-center PW_4_ for the deep desulfurization of fuel. The characterization test results showed that the small MOF-808 nanocrystals grew in the pores and surfaces of SBA-15 to form a complex, MOF-808@SBA-15, and PW_4_ was encapsulated within the pores of MOF-808@SBA-15. The ternary catalyst PW_4_@MOF-808@SBA-15 showed high desulfurization activity and stability in multicomponent model fuel. This is mainly due to three reasons: the high dispersion of the active-center PW_4_ unit, the presence of mesoporous channels promoting the diffusion of reactants and products, and the mesoporous silica carrier improving the stability of the material. Although this ternary catalyst, PW_4_@MOF-808@SBA-15, had an excellent desulfurization effect with a simulated fuel, its application in real fuel needs to be further improved. This new type of ternary composite will open new directions in materials science, as the hybridization of different components will yield more new properties.

## Figures and Tables

**Table 1:** BET surface area, average pore size, and total pore volume of SBA-15, PW_4_@SBA-15, and PW_4_@MOF-808@SBA-15.

Sample	S_BET_ (m²/g)	V (cm^3^/g)	D (nm)
SBA-15	647.16	0.64	3.86
PW_4_@SBA-15	416.29	0.47	4.36
PW_4_@MOF-808@SBA-15	477.05	0.40	3.37

**Table 2:** Comparison of catalytic systems for oxidative desulfurization.

No.	Catalysts	Oxidant	Extractant	Temperature (°C)	Time (min)	Substrate	Removal Efficiency	Refs.
1	PMo_12_@MOF-808@SBA-15	H_2_O_2_	MeOH	70	480	DBT, 4-MDBT, 4,6-DMDBT *	96.8	[35]
2	SRL-POM@MOF-199@MCM-41	O_2_	-	60	150	DBT	100	[36]
3	POM-MOF@Fibercloth	O_2_	-	95	75	DBT	100	[37]
4	PW_11_@MOF-808	H_2_O_2_	MeCN	60	30	DBT	100	[38]
5	(PW_11_Ti)_2_OH@TM-SBA-15	H_2_O_2_	MeCN	70	60	BT, DBT, 4-MDBT, 4,6-DMDBT *	91%	[39]
6	HPMo/SBA-15	H_2_O_2_	-	60	330	DBT	100	[40]
7	HPWA-SBA-15	t-BuOOH	-	50	120	DBT	98.4	[41]
8	PW_4_@MOF-808@SBA-15	H_2_O_2_	MeCN	70	60	DBT, 4-MDBT, 4,6-DMDBT *	99.7	This work

* The model fuel contained several sulfides at the same time.

## Data Availability

The original contributions presented in the study are included in the article, further inquiries can be directed to the corresponding author.
